# A novel online interprofessional education with standardised family members in the COVID-19 period

**DOI:** 10.5116/ijme.6043.8be0

**Published:** 2021-03-26

**Authors:** Mina Suematsu, Noriyuki Takahashi, Kentaro Okazaki, Etsuko Fuchita, Akira Yoshimi, Manako Hanya, Yukihiro Noda, Keiko Abe, Masafumi Kuzuya

**Affiliations:** 1 Department of Education for Community-Oriented Medicine, Nagoya University Graduate School of Medicine, Nagoya, Japan; 2 Department of Integrated Health Sciences, Gerontological Nursing, Nagoya University Graduate School of Medicine, Japan; 3 Division of Clinical Sciences and Neuropsychopharmacology, Faculty and Graduate School of Pharmacy, Meijo University, Nagoya, Japan; 4 Office of Clinical Pharmacy Practice and Health Care Management, Faculty of Pharmacy, Meijo University, Nagoya, Japan; 5 Clinical Nursing, Aichi Medical University College of Nursing, Nagakute, Japan; 6 Department of Community Healthcare & Geriatrics, Nagoya University Graduate School of Medicine, Japan

**Keywords:** COVID-19 period, simulated family member, online interprofessional education, asynchronous and synchronous learning, virtual clinical practice curriculum

## To the Editor

In response to the COVID-19 pandemic, initiatives and guidelines have been suggested for migrating medical education to an online platform worldwide.^1^ Evans and colleagues reported the effectiveness of online interprofessional education (IPE) in improving students' attitudes about interprofessional performance.^2^ Using this approach, we created a new online IPE based on our experience of face-to-face IPE.^3^ The characteristics of our IPE included a clinical scenario with standardised patients (SPs) and standardised family members (SFMs). According to Smithson, SPs play a significant role in healthcare curricula by providing consistency in training scenarios, cultivating a safe space for practice, and providing performance feedback.^4^ For this online IPE, we selected IPE with SFMs because the patient setting in the scenario was people with dementia. Lorin reported that SPs could be trained as SFMs to enhance students' learning, particularly in communicating topics that are difficult to understand.^5^ There were four learning objectives of this IPE. First of all, students would understand their perception of and roles in their own profession and others. Second, they would acquire appropriate attitudes and skills for team communication. Third, they would understand the importance of patient-centred medicine and care. Finally, they would strive to understand how to practice medicine for patients and families as part of a team. This online IPE was a blended learning programme with both asynchronous self-study using online videos and synchronous online discussions modalities that enable real-time participation in small-group meetings.^6^ We used Zoom, a cloud-based video conferencing service for synchronous web conference.

An overview of this programme follows. Teams of medical, nursing and pharmacy students interviewed an SFM whose mother-in-law was hospitalised for treating diabetes. This programme comprised 2 group work sessions and 1 SFM interview session. A 2.5-hour live web conference was provided using the same online platform for both educators and learners. As an asynchronous online modality to promote students' engagement, the following materials were provided: the instruction for using an e-learning system among the three departments and across two institutions, learning outcomes of the IPE programme, knowledge about each profession's roles and perceptions, scenarios of an older patient population with diabetes and dementia, and two 20-minute video lectures about IPE and fundamental information on dementia care from each profession. From 29 May to mid-September 2020, we conducted eight online IPE programmes. Students were divided into two mixed professional groups; each group consisted of 3–4 medical, three nursing, and one pharmacy student for a total of 44 medical, 40 nursing and, 16 pharmacy students; in addition, 6 SFMs and 8 IPE faculties participated. The structure of each programme was composed of three sessions. At first, students used a clinical scenario focusing on an older population with diabetes and dementia. Second, they discussed each profession's role and made care plans. Finally, they interviewed SFMs and explained care plans to SFMs as a mixed professional team during the live web conference. The main roles of SFMs were as follows. SFMs first acted as family members who lived with the scenario of a patient with dementia. Second, SFMs were interviewed by students. Finally, SFMs gave feedback to the students to promote their reflection. We asked the students to describe the most important thing they learnt regarding interprofessional collaboration after finishing the programme.

Results showed several specific problems to resolve. First, addressing technical issues and skills was critical, such as internet connectivity-troubleshooting for all participants and operating the system for breakout sessions for small-group discussions. Second, in contrast to face-to-face IPE facilitation experiences, IPE faculties were more often called on to facilitate students to promote discussion for group work, which was attributed to students feeling nervous and taking more time to become accustomed to talking with others via web conference. Third, SFMs initially experienced some difficulties in giving students feedback online, noting vague eye contact and body language. After resolving these issues, the programme moved forward and generally met students' learning objectives compared with previous face-to-face IPE.

In our experience, it was essential that our IPE team comprising faculty with SFMs discussed ways to provide enhanced online IPE programmes for undergraduate students through web conference before and after each programme. This helped us achieve greater levels of interprofessional collaboration as an IPE team for curricular development. It would be a good learning experience for students if the faculty members demonstrate a deep commitment to interprofessional collaboration. Our challenge of developing an online IPE programme with SFMs may help many educators who are considering a virtual clinical practice curriculum in COVID-19 period. Future research is needed to compare the learning effectiveness of online IPE with that of our previous face-to-face IPE.

### Acknowledgments

This work was supported by JSPS Grants-in-Aid for Scientific Research Grant Number JP 18K02060. Proofreading and editorial assistance were provided by Dr Sundari Joseph and Prof Lesly Diack.

### Conflict of Interest

The authors declare that they have no conflict of interest.
